# Pairing sound with vagus nerve stimulation modulates cortical synchrony and phase coherence in tinnitus: An exploratory retrospective study

**DOI:** 10.1038/s41598-017-17750-y

**Published:** 2017-12-11

**Authors:** Sven Vanneste, Jeffrey Martin, Robert L. Rennaker, Michael P. Kilgard

**Affiliations:** 1 Lab for Clinical and Integrative Neuroscience, School for Behavioral and Brain Sciences, University of Texas at Dallas, Richardson, TX USA; 2 Callier Center of Communication Disorders, School for Behavioral and Brain Sciences, University of Texas at Dallas, Richardson, TX USA; 3 Texas Biomedical Device Center, University of Texas at Dallas, Richardson, TX USA

## Abstract

Recent research has shown that vagus nerve stimulation (VNS) paired with tones or with rehabilitative training can help patients to achieve reductions in tinnitus perception or to expedite motor rehabilitation after suffering an ischemic stroke. The rationale behind this treatment is that VNS paired with experience can drive neural plasticity in a controlled and therapeutic direction. Since previous studies observed that gamma activity in the auditory cortex is correlated with tinnitus loudness, we assessed resting-state source-localized EEG before and after one to three months of VNS-tone pairing in chronic tinnitus patients. VNS-tone pairing reduced gamma band activity in left auditory cortex. VNS-tone pairing also reduced the phase coherence between the auditory cortex and areas associated with tinnitus distress, including the cingulate cortex. These results support the hypothesis that VNS-tone pairing can direct therapeutic neural plasticity. Targeted plasticity therapy might also be adapted to treat other conditions characterized by hypersynchronous neural activity.

## Introduction

Since the first human vagus nerve stimulation (VNS) implantation in 1989, more than 100,000 patients with medical or surgical refractory epilepsy have been treated with VNS worldwide^1^. The rationale behind the treatment is that VNS triggers the release of neuromodulators in the brain that induce an antiepileptic effect. More recently, it was suggested that VNS paired with experience can drive plasticity in a more controlled and therapeutic direction that may direct plasticity in order to treat many manifestations of neurological disorders^2–6^. Indeed, recent research has shown that VNS paired with tones or with rehabilitative training can help patients suffering from tinnitus or undergoing motor rehabilitation after ischemic stroke^2,3,6^.

Anatomical, electrophysiological, and biochemical findings suggest that VNS engages the cholinergic and noradrenergic neuromodulatory systems and may even drive the NMDA receptor and GABAa receptor expression levels, thereby influencing neuronal excitability^7^. In a recent paper, it was shown that pairing VNS with tones can indeed drive neural plasticity and reverse the behavioral correlate of tinnitus in noise-exposed rats^4,8,9^. In a first set of experiments, Engineer and colleagues were able to show that pairing a single pure tone with VNS is sufficient to generate specific and long-lasting changes in the cortical map^4^. In a second set of experiments, they were able to show that repeatedly pairing a range of tone frequencies (but not the tinnitus frequency) with VNS can be used to reverse the behavioral and neural correlates of tinnitus in noise-exposed rates. A reduction in synchronization at the auditory cortex was observed in noise-exposed rats after VNS/multiple tone pairing after treatment similar to control animals, while the sham treatment group had an increase in synchronization compared to the control and treatment groups^4^.

Previous human research using EEG^10,11^, MEG^12,13^, fMRI^14^, and PET^15^ have shown that tinnitus is associated with hyperactivity in the auditory cortex. In tinnitus patients, changes in resting state MEG and EEG measurements over the temporal cortex go together with a reduction in alpha power (8–12 Hz) and increases in theta (3.5–7.5 Hz) and gamma power (>30 Hz) in this area^10,12,13,16^. A resting state EEG study has further demonstrated that the amount of gamma-band activity in the auditory cortex reflects subjective tinnitus loudness, while no effect could be obtained looking at psychoacoustic measures^10,17,18^. Reduction of the subjective tinnitus loudness through transcranial magnetic stimulation^19^ or acoustic coordinated reset neuromodulation (a specific form of auditory stimulation with the aim to desynchronize hypersynchronous activity in the auditory cortex)^11^ is associated with a normalization of gamma power. A recent study further demonstrated that after reduction of subjective tinnitus loudness, the pathological alpha- and gamma-frequency activity reverses^16^, while worsening of tinnitus can increase the activity in the gamma frequency^20,21^.

Tinnitus treatment options are as diverse as their outcomes. Both pharmacological and non-pharmacological modalities are used with only limited success^22–24^. Psychological treatment tends to improve the emotional symptoms that go together with the tinnitus percept^25^. However, a group of patients remains refractory to these treatments^26^. For various pathologies, this is the point in which surgical treatments like neuromodulation find their place^26^. One new treatment option could be neuromodulation for patients intractable to conservative medical practice. In this study, we investigate if pairing VNS with tones could drive plasticity in the auditory cortex of humans for the first time. We assess the effect of VNS paired with tones on changes in synchronization in patients with tinnitus by investigating resting-state source-localized EEG before and after VNS treatment. We hypothesized that the behavioral improvement caused by VNS paired with tones would be associated with a decrease in synchronization in the gamma frequency band and an increase in synchronization in the alpha frequency band in the auditory cortex. The goal of this study was to better understand the underlying neural mechanisms associated with the therapeutic response to VNS-tone pairing.

## Methods

### Participants

Eighteen patients with tinnitus were included in this study. Ten participants came from the Antwerp site, while eight patients came from the Dallas site. Patients’ characteristics can be found in Table 1. The trial for Antwerp site was registered on clinicaltrials.gov (NCT01253616; date of submission: December 2, 2010), while the Dallas site was part of a multicenter study on clinicaltrials.gov (NCT01962558; date of submission: October 9, 2013). Both sites had IRB approvals. For the Antwerp data, this study was approved by Antwerp University Hospital Ethics Committee and then reviewed by the Belgian Competent Authority. For the Dallas data, this study was approved by the office of research compliance at the University of Texas at Dallas and the FDA Investigational Device Exemption (IDE, #G130140). All participants gave written informed consent. The clinical trial data for Antwerp were published in Neuromodulation^3^. The data from the Dallas site are part of a prospective double-blind randomized controlled multicenter study published in scientific reports^27^. Neither study reports electrophysiological data. For the multicenter study, only the Dallas site collected electrophysiological data. We carried out the study in accordance with the approved guidelines. Data were collected in the context of a VNS clinical trial. However, the data in current paper are retrospectively analyzed.

**Table 1:** Baseline demographics (mean and standard deviation).

**Baseline characteristic**	**Antwerp (n = 10)**	**Dallas (n = 8)**
Age (yrs.)	45.6 (9.0)	54.9 (9.1)
Gender	Male: 8/Female: 2	Male: 6/Female: 2
Tinnitus duration (yrs.)	5.4 (4.10)	19.2 (15.9)
Tinnitus pitch (kHz)	8.8 (4.1)	9.6 (2.71)
Loudness Match (dB)	53.4 (20.32)	63.5 (24.47)

### VNS parameters and procedure

All patients had an implanted VNS device (MicroTransponder Inc., Austin, TX; see supplementary materials). The patients in the Antwerp study had an external controller system which consisted of a commercial laptop computer with headphones that communicated with a commercially available external stimulator (World Precision Instruments-DS8000) to deliver VNS pulses. The patients in the Dallas study had an external controller system which consisted of a commercial laptop computer with headphones running the Model 4000 Tinnitus Application Programming Software (TAPS) connected to an external controller that communicates with the internal pulse generator (IPG) via wireless transmitter (WT). The external system synchronized tones with VNS to provide investigator control of settings for both the IPG stimulator and tones.

Each VNS simulation consisted of fifteen 0.8-mA, constant-current, charge-balanced pulses (100-μs pulse width, 30 Hz frequency). The duration of the VNS pulse was 0.5 seconds. Each pulse train was delivered approximately every 30 seconds for 2.5 hours; the exact timing was randomized to provide enough variability such that the patient could not guess exactly when stimulation would occur. We used the exact same parameters as in the original animal study, including this randomization. All stimulation parameters were based on earlier animal and human studies^3,4]^.

First, a pitch matching procedure was used to identify the most prominent pitch of the subject’s tinnitus. The audiologist started with a 1000-Hz pulsed tone and used a bracketing procedure. The presentation level was adjusted to about the same loudness as the tinnitus. We presented a tone and asked the patient, “Tell me if the most prominent pitch of your tinnitus is higher or lower than the pitch of my tone”. If the patient said higher, we went up by half an octave, and if they say lower, we went down by half an octave. The run was stopped when the tinnitus pitch was bracketed and the last test frequency was recorded. This was repeated 7 times and the average taken as the most prominent tinnitus pitch.

In the Paired VNS group, each 0.5-second VNS pulse was presented simultaneously with a 0.5-second tone. Subjects heard several randomly interleaved tones during the therapy and each tone was paired with VNS but excluded one or more of the subject’s tinnitus frequencies. The tones paired with VNS were at least a half octave away from the most prominent tinnitus pitch for each individual subject. The frequencies ranged from 125–12500 Hz in half-octave steps and were played at an intensity based on the patient’s comfort level and adjusted for any hearing loss at different frequencies. The frequency and intensity (in dB HL) of each tone were randomly selected each time a VNS pulse was delivered. Each of the tone frequencies were made to appear to arise from various 3D locations (programmed using a KEMAR head model) in order to avoid a bias of presenting a tone (paired with VNS) from a single spatial location. For each frequency, the tone intensity was based on the patient’s audiogram. If the threshold exceeded 40 dB HL, the intensity of the tone delivered was 80 dB HL. For thresholds between 20–40 dB HL, the tone intensity was 70 dB HL. For thresholds 0–20 dB HL, the tone intensity was set to 60 dB HL. Any tone that would be presented above 80 dB SPL using these criteria was limited to 80 dB SPL. Stimulation was delivered to the left vagus nerve since this is the most common practice in VNS for epilepsy and depression. However, since the upstream targets are bilateral, stimulation should affect both of the cerebral hemispheres.

Subjects in the Belgium study received treatment for 2.5 hours/day for 4 weeks, while subjects in the Dallas study received the treatment for 2.5 hours/day for 6 (n = 4) or 12 (n = 4) weeks, depending on whether subjects were assigned first to the control condition (sham) or not. The sham patients received VNS for 6 weeks for 2 hours and 15 minutes before and after the VNS tones. After 6 weeks, these patients received paired VNS treatment.

Patients were instructed to be in a quiet room during therapy and to read a magazine, book, etc. while sitting in a comfortable chair. The patients were also instructed not to sleep or have extended conversations during therapy; however, they were allowed to work on a muted computer. The intent was to allow the patient to hear the tones while still being able to perform some other tasks.

### Outcome measures

To determine tinnitus loudness, participants were asked to self-rate loudness on a 0–100 visual analogue scale; 0 meaning no tinnitus and 100 indicating the loudest tinnitus that they can imagine. This estimation was performed either for both ears or, in cases of unilateral tinnitus, documented as only occurring in one ear.

The Tinnitus Handicap Inventory (THI) was selected for implementation because it is a brief and easy-to-administer questionnaire that is suitable for use in busy clinical settings^28^. The THI is a 25-item self-administered questionnaire that aims to quantify the impact of tinnitus on quality of life by measuring its effects on everyday function. Respondents are asked to answer the questions with ‘Yes’ (4 points), ‘Sometimes’ (2 points), or ‘No’ (0 points). A higher tinnitus handicap questionnaire score (maximum 100) is indicative of a greater tinnitus handicap.

### Electrophysiological recordings

As a standard procedure EEG data were obtained before and immediately after the last sessions of VNS-treatment (post). The EEG recordings were obtained in a fully lighted room and lasted approximately five min.Each participant was sitting upright on a small but comfortable chair. For the Belgium population we used a Mitsar-201 amplifiers (http://www.novatecheeg.com/) with 19 electrodes placed according to the standard 10–20 International placement.For the Dallas population we used Neuroscan Symaps amplifiers (http://compumedicsneuroscan.com/) with 64 electrodes placed according to the standard 10–10 International placement. Impedances were checked to remain below 5 kΩ. Data were collected eyes-closed (sampling rate = 500 Hz, band passed 0.15–200 Hz). Off-line data were resampled to 128 Hz, and band-pass filtered in the range 2–44 Hz. We plotted data and carefully inspected for manual artifact-rejection. We removed all episodic artifacts including eye blinks, eye movements, teeth clenching, body movement, or EEG artifact from the stream of the EEG. As different EEG systems, different environmental noise sources, different hardware filters can generate different signals between the two sites (Antwerp–Dallas), we checked our data by providing a cross-frequency analysis in both groups showing that there is no significant difference between the two groups (see supplementary material).

An average Fourier cross-spectral matrices wascomputed including delta (2–3.5 Hz), theta (4–7.5 Hz), alpha (8–12 Hz), low beta (13–21 Hz), high beta (21.5–30 Hz), and gamma (30.5–44 Hz) as the frequency bands. We used standardized low-resolution brain electromagnetic tomography (sLORETA) to estimate the intracerebral electrical sources using a common average reference transformation^29^. sLORETA computes electric neuronal activity as current density (A/m^2^) without assuming a predefined number of active sources. The solution space used in this study and associated lead-field matrix are those implemented in the LORETA-Key software (http://www.uzh.ch/keyinst/loreta.htm). In addition, the log-transformed electric current density was averaged across all voxels belonging to the region of interest (left auditory cortex) for the different frequency bands.

Lagged phase coherence between two sources was calculated By extracting the time-series of current density for different regions of interests using sLORETA. Power in all 6,239 voxels was normalized to a power of 1 and log-transformed at each time point so that the region-of-interest values reflect the log-transformed fraction of total power across all voxels, separately for specific frequencies. Regions of interest selected were the left and right auditory cortex, the left and right parahippocampus, the left and right insula, the dorsal anterior cingulate cortex, the subgenual anterior cingulate cortex, and the posterior cingulate cortex. These regions of interest were selected based on the previous tinnitus literature^30^.

### Statistical analysis

#### Behavioral measures

A comparison was made between pre and post VNS treatment on the visual analogue scale for loudness and for the tinnitus handicap inventory using a paired *t*-test. A Pearson correlation was calculated between the visual analogue scale for loudness and the tinnitus handicap inventory for pre-VNS treatment and post-VNS treatment.

#### Statistical analyses on the whole brain

We use a non-parametric permutation methodology. This method is based on estimating, via randomization, the empirical probability distribution for the max-statistic under the null hypothesis comparisons^31^. This methodology corrects for all voxels and for all frequency bands. As this method is non-parametric in nature, the assumption of Gaussianity is not nescessary^31^. The significance threshold for all tests was based on 5000 permutations. Comparisons were made between the pre-VNS and post-VNS treatment. These comparisons were performed on a whole brain by sLORETA statistical contrast maps through multiple voxel-by-voxel comparisons in a logarithm of *F*-ratio.

#### Region of interest analysis

A comparison was made between pre- and post-VNS treatment for the log-transformed current density for the theta, alpha, and gamma frequency bands using a paired *t*-test. We selected these frequency bands because previous research already indicated changes in these frequency bands within the auditory cortex. A Pearson correlation was calculated between the visual analogue scale and the log-transformed current density of the auditory cortex at gamma frequency at baseline and after VNS treatment as well as between pre and post VNS treatment. A similar analysis was applied for the THI.

#### Statistical analyses for the lagged phase coherence

We cacluated the lagged phase contrast maps and correlated with the visual analogue scale for loudness and the THI, respectively, for each frequency bands. Similar to the whole brain analysis we based ourt he significance level on a permutation test with 5000 permutations. This methodology corrects for all voxels and for all frequency bands.

## Results

### Behavioral results

**Figure 1:**
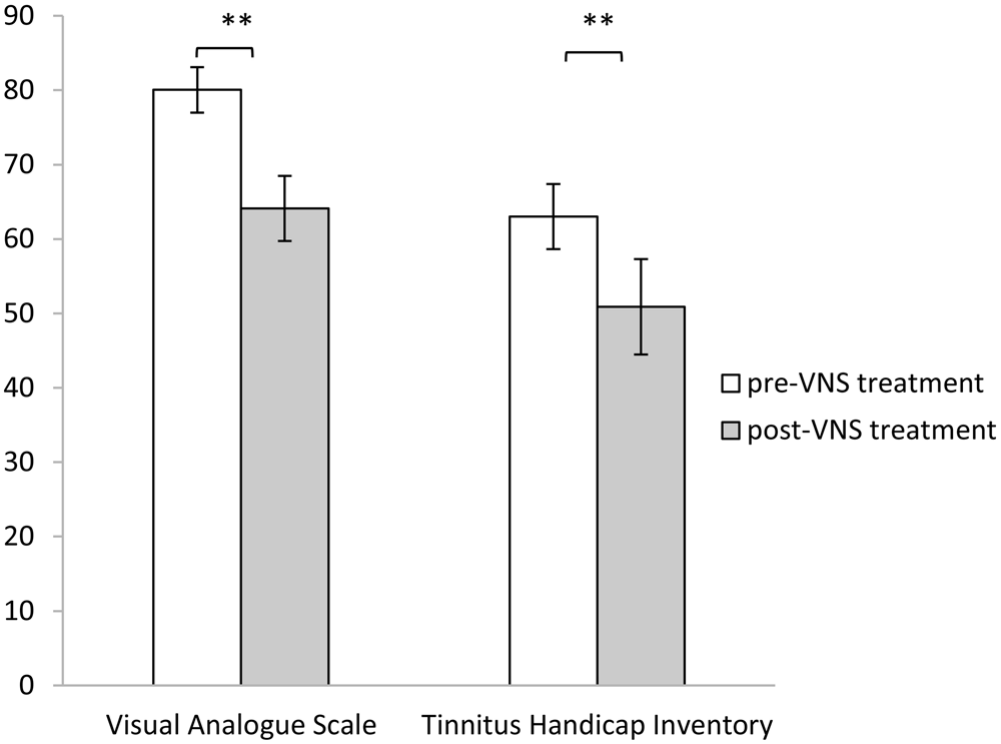
A comparison between pre and post VNS treatment for the visual analogue scale for loudness and the tinnitus handicap inventory shows a significant effect for both scales (**p ≤ 0.01).

**Figure 2:**
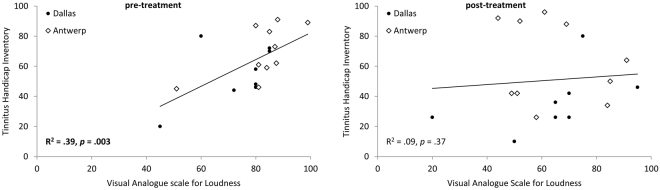
Pearson correlation between the visual analogue scale for loudness and the tinnitus handicap inventory, before and after the VNS treatment. (Dots represent participants from the Dallas group, while diamonds represent participants from the Belgium group).

For both the loudness (measured with the VAS; *t* = 2.72, *p* = 0.01) and emotional (measured with the THI; *t* = 3.32, *p* = 0.004) components of the tinnitus percept, we observed a significant reduction after VNS treatment in comparison to before VNS treatment (see Fig. 1). On average, the loudness component was reduced from 80.06 (*Sd* = 12.97) to 64.10 (*Sd* = 18.5) (reduction of 18.19%). The emotional component was reduced from 63.00 (*Sd* = 19.30) to 50.89 (*Sd* = 27.19) (reduction of by 19.22%). The correlation between the pre-VNS loudness and the emotional component was significant and positive (Fig. 2A, r = 0.62, *p* = 0.003). After VNS treatment, there was no longer a significant correlation between loudness and the emotional component (Fig. 2B, r = 0.30, *p* = 0.37). See Table 2 for the individual scores.

**Table 2:** Patient scores on THI and VAS.

**Site**	**Patient No**.	**Stimulation weeks**	**VAS**		**THI**	
			**Pre**	**Post**	**Pre**	**Post**
Antwerp	1	4	51	58	45	26
Antwerp	2	4	87	84	73	34
Antwerp	3	4	84	91	59	64
Antwerp	4	4	88	69	91	88
Antwerp	5	4	88	85	62	50
Antwerp	6	4	81	51	61	42
Antwerp	7	4	85	61	83	96
Antwerp	8	4	80	44	87	92
Antwerp	9	4	99	52	89	90
Antwerp	10	4	81	49	46	42
Dallas	1	6	80	20	58	26
Dallas	2	12	72	65	44	36
Dallas	3	6	90	95	80	46
Dallas	4	6	80	65	48	26
Dallas	5	12	85	75	72	80
Dallas	6	12	45	50	20	10
Dallas	7	6	85	70	70	42
Dallas	8	12	80	70	46	26
Total	Mean		80.06	64.10	63.00	50.89
	Sd		12.97	18.58	19.30	27.19

THI: Tinnitus Handicap Inventory. VAS: Visual Analogue Scale.

### Electrophysiological results

**Figure 3:**
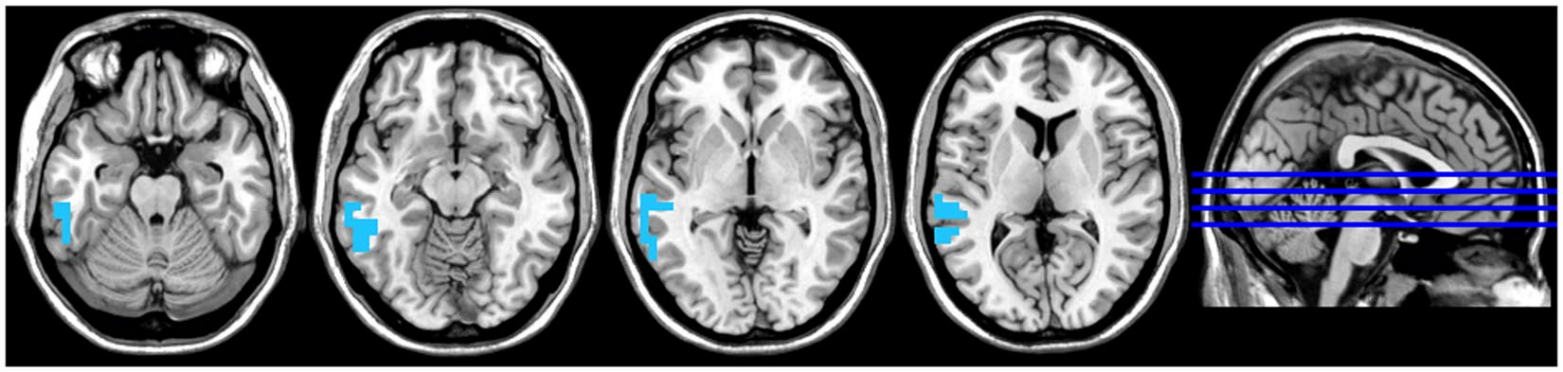
A whole brain analysis showed a significant effect for the gamma frequency band indicating a decrease in synchronized activity at the left auditory cortex after VNS treatment in comparison to before treatment.

A whole brain analysis showed a significant effect for the gamma frequency band, indicating a decrease in synchronized activity in the left auditory cortex extending into the middle and inferior temporal gyri after VNS treatment compared to before treatment (see Fig. 3). No significant effect was obtained for the delta, theta, alpha, low beta, or high beta frequency bands.

**Figure 4:**
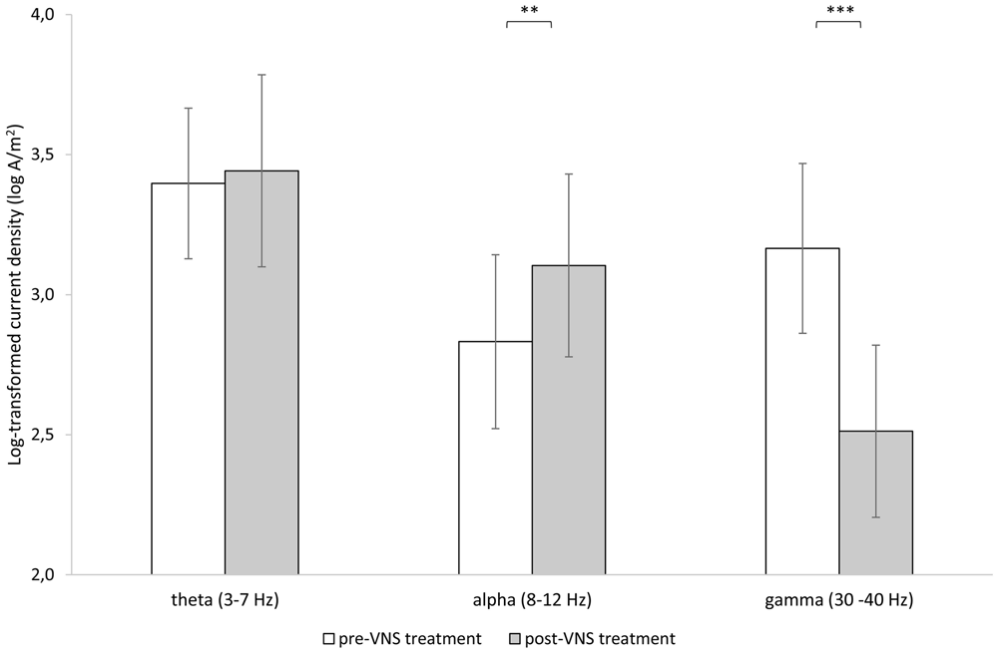
A region of interest for the left auditory cortex showed a significant effect for the alpha and gamma frequency bands, but not for the theta frequency band when comparing pre and post VNS treatment. (**p < 0.01; ***p < 0.001).

A region of interest analysis for the left auditory cortex showed significant effects for the alpha (*t* = −2.81, *p* = 0.012) and gamma (*t* = 3.79, *p* = 0.001) frequency bands, but not for the theta (*t* = −21, *p* = 0.83) frequency band (see Fig. 4). For the alpha frequency band, increased synchronized activity was observed after VNS treatment in comparison to before VNS treatment. For the gamma frequency band, decreased synchronized activity was observed after VNS treatment in comparison to before VNS treatment.

**Figure 5:**
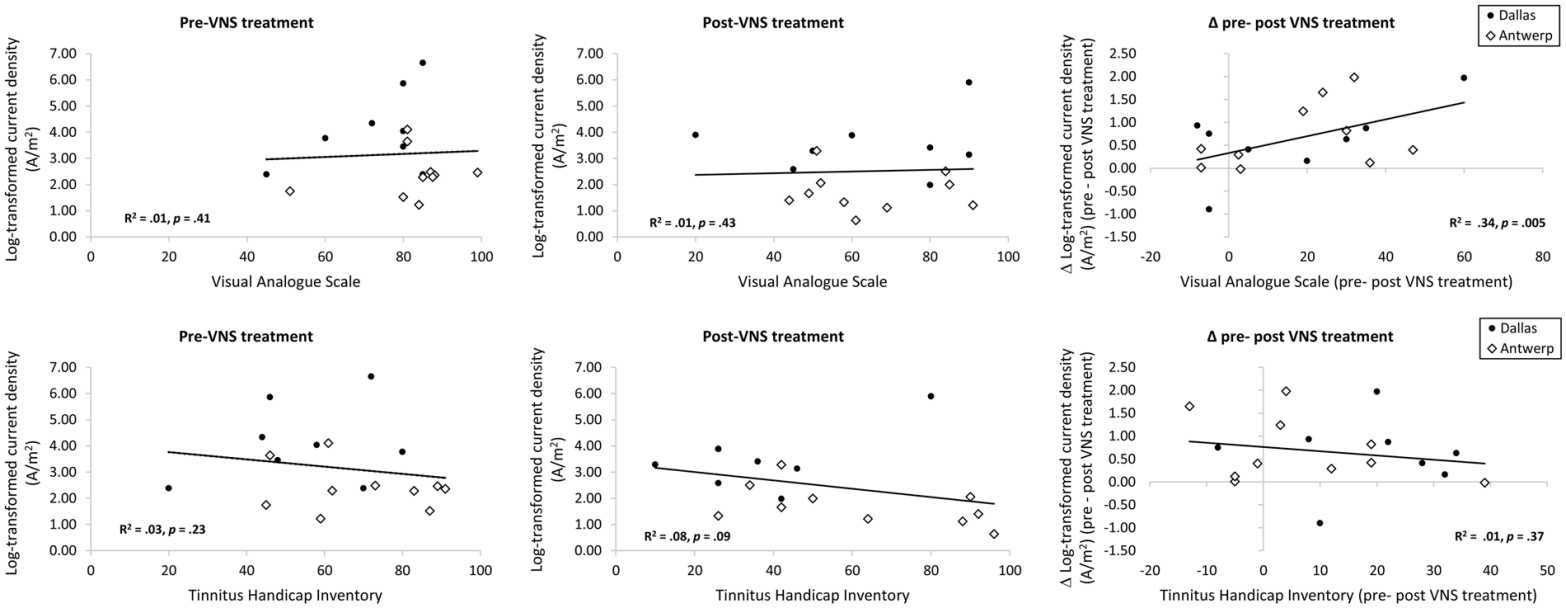
Top: A correlation analysis between the visual analogue scale and the log-transformed current density of the auditory cortex at gamma frequency at baseline and after VNS treatment as well as the difference between pre and post VNS treatment. Bottom: A correlation analysis between Tinnitus handicap inventory and the log-transformed current density of the auditory cortex at gamma frequency at baseline and after VNS treatment as well as the difference between pre and post VNS treatment. (Dots represent participants from the Dallas group, while diamonds represent participants from the Belgium group).

A correlation analysis revealed that reduction in loudness correlates positively with reduction in gamma synchronization in the left auditory cortex (r = 0.58, *p* = 0.005). There was not a significant correlation between the visual analogue scale for loudness and the log-transformed current density of the auditory cortex at gamma frequency at baseline (r = 0.06, *p* = 0.41) and after VNS treatment (r = −0.05, *p* = 0.43). A correlation analysis for the emotional component of the tinnitus and the log-transformed current density of the auditory cortex at gamma frequency revealed no significance at baseline (r = −0.18, *p* = 0.23), after VNS treatment (r = −0.33, *p* = 0.09), or in the changes between pre and post treatment (r = −0.08, *p* = 0.37). See Fig. 5d–e. No significant correlations were observed for the theta or alpha frequency bands.

**Figure 6:**
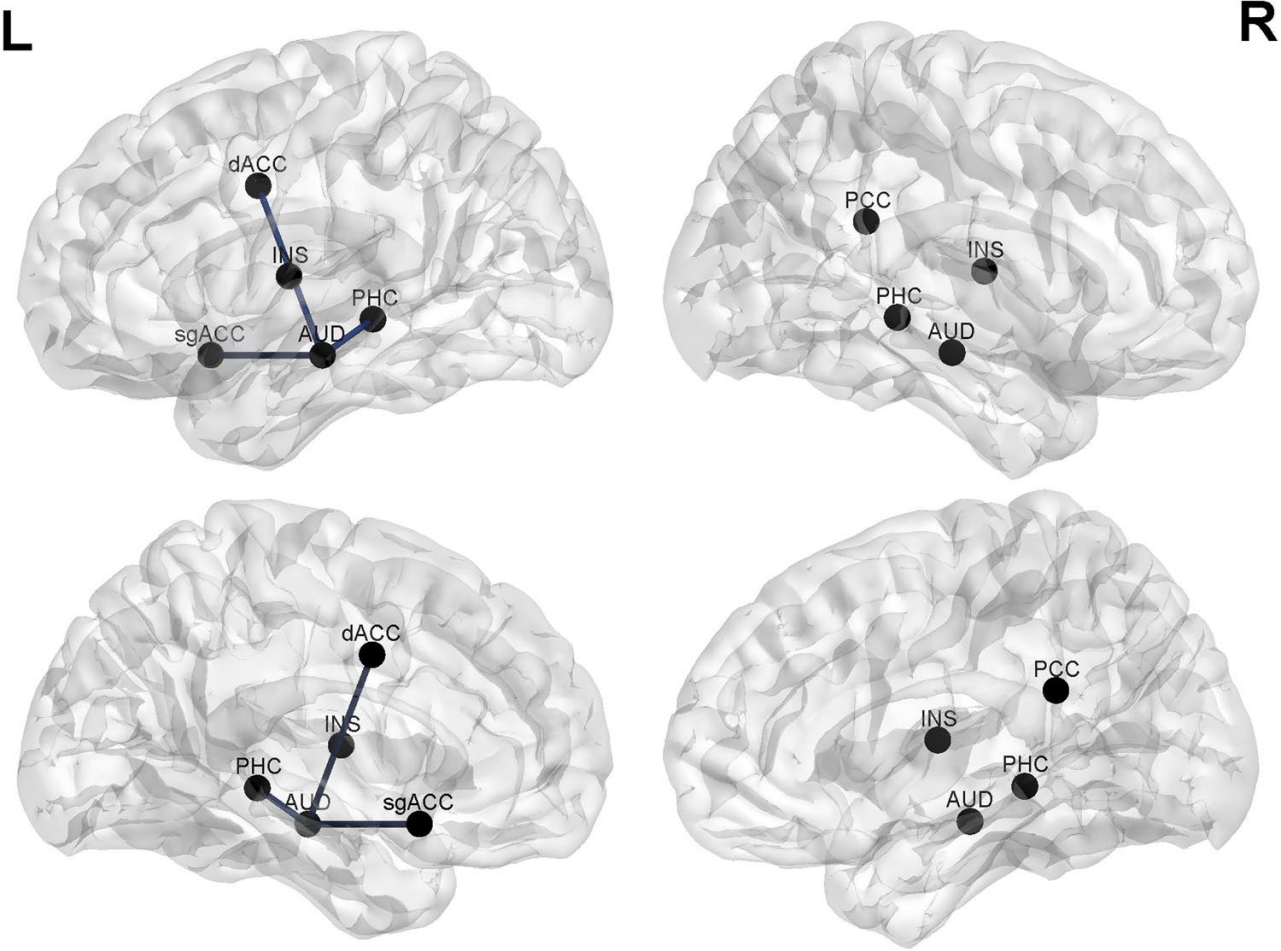
A connectivity analysis revealed a significant effect for the theta frequency, indicating a reduction after VNS treatment compared to pre-treatment in lagged phase coherence between the auditory cortex and the dorsal anterior cingulate cortex and the subgenual anterior cingulate cortex and the left parahippocampus respectively.

An analysis of phase coherence revealed a significant effect (r = 0.42, *p* < 0.05) for the theta frequency, indicating a reduction after VNS treatment in comparison to pre-treatment in lagged phase coherence between the auditory cortex and the dorsal anterior cingulate cortex and the subgenual anterior cingulate cortex and the left parahippocampus respectively (see Fig. 6). No significant effects were obtained for the delta, alpha, low beta, high beta, or gamma frequency bands.

**Figure 7:**
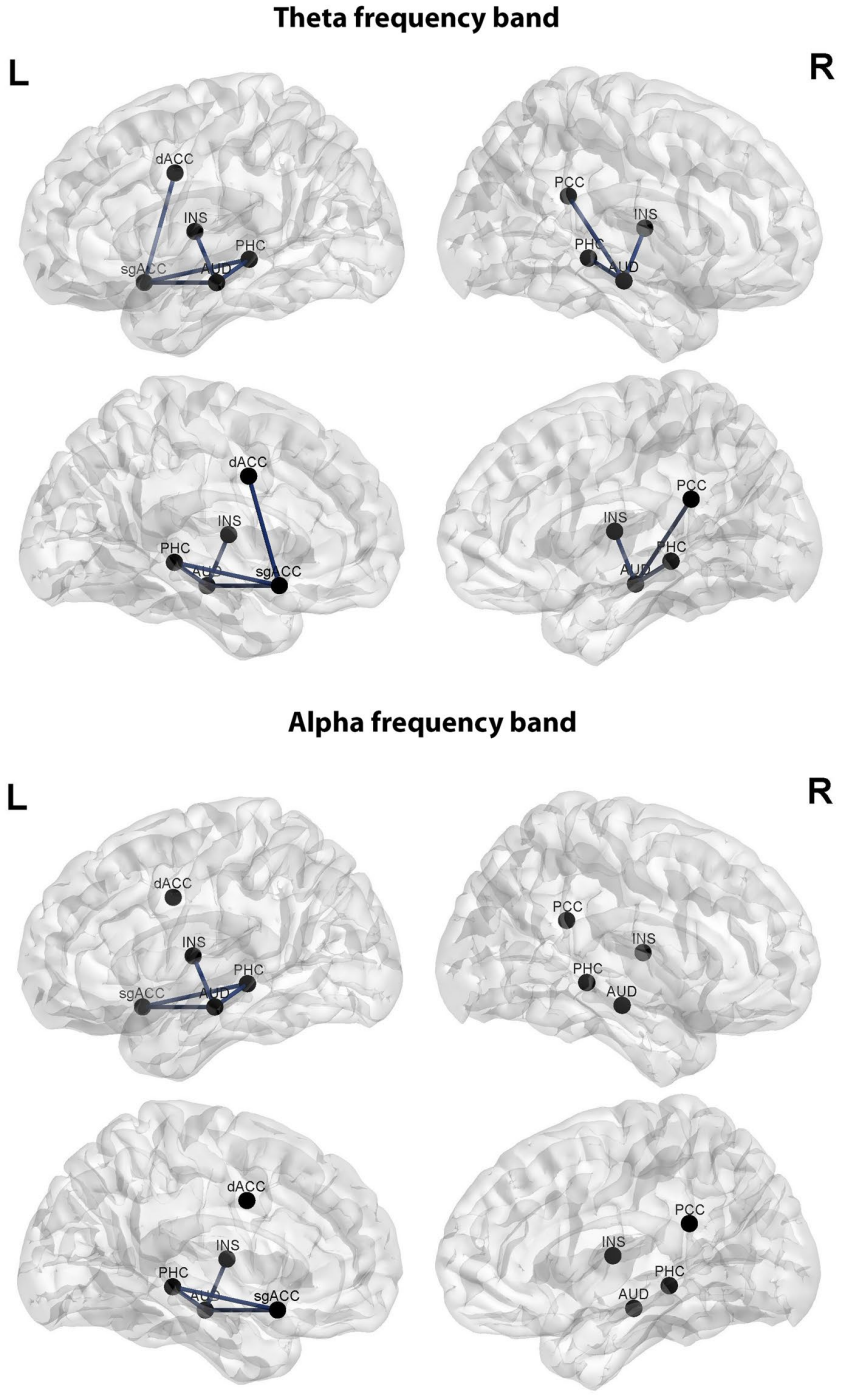
Connectivity analysis looking at the difference in pre versus post treatment connectivity and the difference on the visual analogue scale for loudness pre versus post treatment revealed a significant effect for the theta and alpha frequency bands.

Analysis of the difference in phase coherence pre versus post treatment and the difference in the THI pre versus post treatment revealed no significant effect for the delta, theta, alpha, low beta, high beta, or gamma frequency bands. A similar analysis for the visual analogue scale for loudness revealed a significant effect for the theta (r = 0.34, *p* < 0.05) and alpha frequency bands (r = 0.31, *p* < 0.05). For both frequency bands, we observed a reduction in lagged phase coherence between the auditory cortex and the subgenual anterior cingulate cortex and the left parahippocampus respectively, which is associated with a reduction on the loudness scale (see Fig. 7).

## Discussion

This study provides the first human evidence in support of the hypothesis that pairing VNS with experience drives neural plasticity associated with a reduction in the tinnitus percept. More precisely, we observed that VNS paired with tones desynchronized the left auditory cortex of tinnitus patients at the gamma frequency band. The reduction in gamma frequency band activity was correlated with the amount of loudness reduction. VNS paired with tones also increased synchronization in the left auditory cortex at the alpha frequency band. Although theta frequency synchronization does not seem to change after VNS paired with tones, we observed a reduction in the phase coherence between the left auditory cortex and respectively the dorsal anterior cingulate cortex and the subgenual anterior cingulate cortex and the parahippocampus that is associated with loudness suppression. A similar effect was obtained for the alpha frequency band. The reduction of the emotional component of tinnitus after VNS treatment was not associated with any of the neural measures recorded.

Our results are consistent with previous reports that reduction of tinnitus loudness is associated with desynchronization of hypersynchronous activity in the auditory cortex at the gamma frequency band^16,19^. Our results are also consistent with the findings in animals that VNS-tone pairing reverses the increased in cortical synchronization observed in noise-exposed rats^8^. The amount of gamma-band synchronization in the human auditory cortex is correlated with subjective tinnitus loudness^10^. We were not able to show that gamma activity correlates with perceived tinnitus loudness before the VNS treatment, but demonstrated that the amount of decreased synchronization at the gamma frequency band was correlated with the amount of loudness suppression after VNS treatment. Previous research indeed proposed that gamma activity depends on the match between the top-down predictions and bottom-up sensory input, where the gamma activity is modulated as a function of sensory surprise and is used to signal unexpected information, i.e. prediction error^32,33^. It can be hypothesized that this prediction error is reduced due to VNS with tone pairing, reflecting the reduction in synchronized activity in the auditory cortex that is associated with a reduction in tinnitus loudness. Together with gamma reduction, we also observed an increase in the alpha synchronization of the auditory cortex after VNS treatment. This is consistent with previous reports of less pathological alpha and gamma frequency activity after reduction of tinnitus loudness^16^.

Although no effect was obtained for the theta signal before and after the VNS-paired tone treatment, changes were demonstrated in phase coherence for the theta and alpha frequency bands that correlate with changes in the loudness percept. That is, after targeted plasticity therapy we saw a decrease in phase coherence between the auditory cortex and areas that have been associated with the affective components (i.e. distress) of tinnitus, such as the dorsal anterior cingulate cortex and the subgenual anterior cingulate cortex^34^, as well as areas that have been associated with the tinnitus percept, such as the parahippocampus^35,36^. The involvement of these non-auditory areas in relation to emotional factors in tinnitus has previously been reported^34,35,37,38^. It is interesting to note that the original VNS therapy paired with tones was developed to modulate the tinnitus percept and not necessarily the emotional component related to the tinnitus. Our data in this study show a correlation between the loudness and the emotional component before the treatment that does not remain after the VNS therapy. In addition, we see a correlation between the loudness and reduction in gamma synchronization in the left auditory cortex. Taking these findings together, we are probably directly modulating the loudness percept and indirectly the emotional component. This is potentially done by reduced connectivity between emotional related brain areas and the loudness related brain areas.

Research also suggests that these non-auditory areas are associated with the conscious phantom percept, which suggests that the sensory cortices are not sufficient for the generation of a conscious percept and implicate an involvement of these non-auditory areas in conscious perception^39,40^. Accordingly, Jastreboff suggested the more frontal areas as “candidates for the integration of sensory and emotional aspects of tinnitus^41^”. This idea was corroborated by recent studies demonstrating abnormal long-range coupling in tinnitus patients^35,42–45^. Previous research already showed that increased coherence in the alpha frequencies between affective areas and loudness areas of the brain is associated with tinnitus^35^. That we found reduced coherence for the theta and alpha frequency bands suggests either a disintegration of the information or a suppression of the conscious phantom percept and confirms the idea that alpha coherence plays an important role in the tinnitus percept. This corroborates with a decrease in the correlation between the loudness and the emotional component post-VNS treatment in comparison to pre-VNS treatment.

Overall, our findings are consistent with the thalamocortical dysrhythmia hypothesis, which states that in a deafferented state after frequency-specific auditory deprivation (i.e. hearing loss), the dominant rhythm present in normal circumstances (i.e. alpha activity) decreases to theta frequencies in the deafferented thalamocortical column. As a result, GABAa-mediated lateral inhibition is reduced, inducing a halo-shaped region of gamma band activity known as the edge effect^46,47^ and therefore possibly contributing to the perception of a phantom sound. Enhanced synchronization in the gamma frequency band in the auditory cortex is regarded as a direct electrophysiological correlate of auditory phantom perception^13,48^. The fact that suppression of tinnitus after treatment by VNS paired with tones is associated with desynchronization in the auditory cortex at the gamma frequency band suggests the importance of the electrophysiological correlate of the auditory phantom sensation. Although thalamocortical dysrhythmia underlines the importance of pathological gamma frequency oscillations, the impact of reduced alpha oscillations should not be neglected^46,49^. Previous research has suggested that low levels of alpha are associated with a state of excitation, whereas high levels of alpha are associated with a state of inhibition^13,49,50^. The fact that alpha synchronization increases after VNS with tone pairing suggests that the alpha frequency helps to inhibit the percept, which fits with the idea that alpha is involved with frequency-specific auditory deprivation of the dominant rhythm, as suggested by the thalamocortical dysrhythmia concept^12,48^.

Thalamocortical dysrhythmia is the function of GABAa that mediates decreases in inhibition. This has been associated with increased gamma-band synchronization^12,51^. Moreover, it has been shown that synchronization in the gamma range is the result of impaired GABAergic connections^52^. The role of GABAa in tinnitus was recently confirmed in a human study using magnetic resonance spectroscopy demonstrating the association of a significant reduction in auditory cortex GABA concentration with tinnitus loudness^53^. Interestingly, research has revealed that the therapeutic efficacy of VNS is associated with increased GABA-mediated cortical inhibition by GABAa receptor density normalization^54^. As the beneficial outcomes of VNS can be reflected by the up-regulation of GABA receptors, and due to the interdependency between GABA modulation and gamma activity, it is possible that the effect on tinnitus suppression in our study might be directly related to the increase in GABAa receptor density. However, it is also possible that the gamma decrease could be an indirect effect of VNS-directed plasticity that is facilitated by cholinergic and noradrenergic transmission that promotes spectral and temporal response characteristics of the central auditory neurons to restore the normal characteristics of the circuitry and alleviate the percept of tinnitus^7^. That is, VNS paired with the appropriate presentation of tones could drive plasticity via cholinergic and noradrenergic transmission and promote the number of cortical neurons tuned to frequencies other than the tinnitus frequency in order to reduce the overrepresented tinnitus frequency^8^. This explanation is consistent with the normalization of tonotopic maps in noise-exposed animals after VNS-tone pairing^4,55^.

Interestingly, we observe a lateralization effect demonstrating that VNS paired with tones desynchronized the left auditory cortex of tinnitus patients at the gamma frequency band. An ongoing debate within the literature is if tinnitus is always generated unilateral on the left-side or the contralateral auditory cortex. Based on Functional MRI^56,57^, MEG^13,51,58^, and EEG^10,59^ is it assumed that the tinnitus is located in the contralateral auditory cortex^56^, whereas most positron emission tomography (PET) studies suggest tinnitus is always generated in the left auditory cortex^60,61^. Our findings corroborate this latter view. Furthermore, the treatment is designed to selectively target the tinnitus with fits with our finding. However, this could not explain why specific neuromodulators (GABAergic, cholinergic, and noradrenergic systems) influence neuronal excitability only to the hyperactivity side.

A limitation of this study is that we pooled data from two studies each using a different length of treatment. Nevertheless, these results are unique and fit with our hypothesis. Our confidence in the data is increased due to its consistency with animal data that showed a reduction in synchronization at the auditory cortex in noise-exposed rats after treatment using VNS-multiple tone pairing. An important avenue to further explore the mechanisms underlying this therapy would be to look at changes in the GABAergic, cholinergic, and noradrenergic systems. This could help us to further explain the underlying mechanism of action that drives plasticity using VNS.

In conclusion, our results support the hypothesis that VNS-directed plasticity using tones is associated with desynchronization of auditory cortex gamma frequency activity that is directly correlated with the loudness percept, which could be mediated by GABAa receptor density normalization that might be reflected by an increased synchronization in alpha inhibition. The fact that gamma band desynchronization correlates with the outcome of the VNS treatment suggests that inhibition could be potentially used as a biomarker in assessing the efficacy of VNS treatment. The targeted neuroplasticity therapy of VNS paired with tones is not only reflected by local changes in the auditory cortex, but also induces changes in phase coherence between auditory and non-auditory related cortices in the theta and alpha frequency bands that correlate with changes in the loudness percept that might reflect a suppression of a conscious phantom network. However, more research is needed to further explore the underlying mechanism and the long-term potential of the treatment.

## Electronic supplementary material

Supplementary material
