# Supplementary material for: Pairing sound with vagus nerve stimulation modulates cortical synchrony and phase coherence in tinnitus: An exploratory retrospective study
Source: Sci Rep. 2017 Dec 11;7:17345. doi: 10.1038/s41598-017-17750-y (PMC5725594; doi:10.1038/s41598-017-17750-y)

Supplementary material:

Pairing sound with vagus nerve stimulation modulates cortical synchrony and phase coherence in tinnitus: An exploratory retrospective study.

Sven Vanneste, Jeffrey Martin, Robert L. Rennaker & Michael Kilgard

Different EEG systems, different environmental noise sources, probably also different hardware filters can generate different signals between the two sites (Antwerp – Dallas)? Therefore checked our data by providing a cross-frequency analysis in both groups showing that there is no significant difference between the 2 groups.

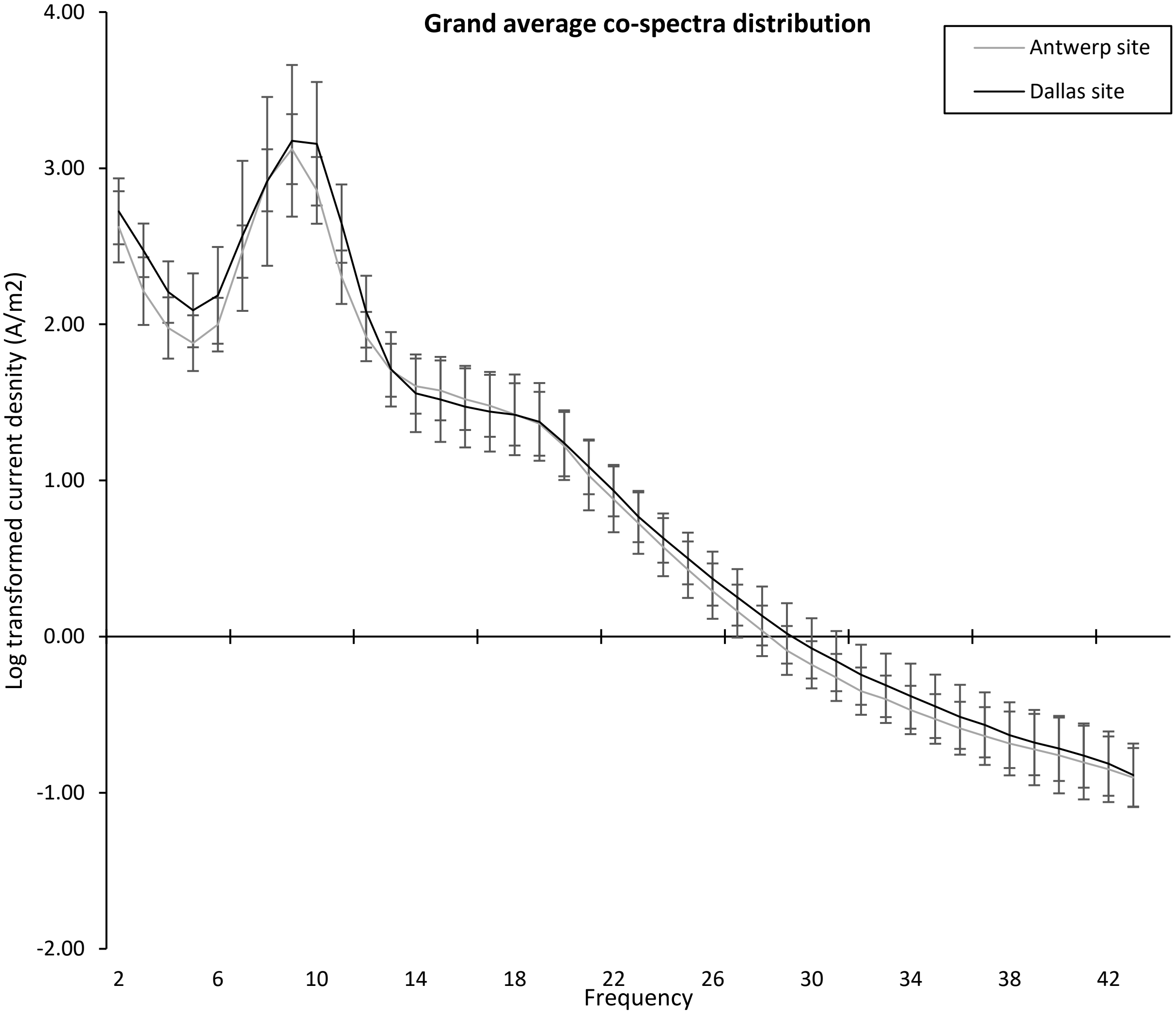

Supplement: Supplementary file 1 — Supplementary material [file 41598_2017_17750_MOESM1_ESM.pdf]
